# Effects of combining exercise with long-chain polyunsaturated fatty acid supplementation on cognitive function in the elderly: a randomised controlled trial

**DOI:** 10.1038/s41598-020-69560-4

**Published:** 2020-07-31

**Authors:** Hisanori Tokuda, Mika Ito, Toshiaki Sueyasu, Hideyuki Sasaki, Satoshi Morita, Yoshihisa Kaneda, Tomohiro Rogi, Sumio Kondo, Motoki Kouzaki, Takashi Tsukiura, Hiroshi Shibata

**Affiliations:** 1 Institute for Health Care Science, Suntory Wellness Ltd., Kyoto, Japan; 2 Safety Science Institute, Suntory MONOZUKURI Expert Ltd., Kyoto, Japan; 3 Medical Corporation Kenshokai, Fukushima Healthcare Center, Osaka, Japan; 4 Laboratory of Neurophysiology, Graduate School of Human and Environmental Studies, Kyoto University, Kyoto, Japan; 5 Department of Cognitive and Behavioral Sciences, Graduate School of Human and Environmental Studies, Kyoto University, Kyoto, Japan

**Keywords:** Health care, Neurology

## Abstract

Multifactorial lifestyle intervention is known to be more effective for ameliorating cognitive decline than single factor intervention; however, the effects of combining exercise with long-chain polyunsaturated fatty acids (LCPUFA) on the elderlies' cognitive function remain unclear. We conducted a randomised, single-masked placebo-controlled trial in non-demented elderly Japanese individuals. Participants were randomly allocated to the exercise with LCPUFA, placebo, or no exercise with placebo (control) groups. Participants in the exercise groups performed 150 min of exercise per week, comprised resistance and aerobic training, for 24 weeks with supplements of either LCPUFA (docosahexaenoic acid, 300 mg/day; eicosapentaenoic acid, 100 mg/day; arachidonic acid, 120 mg/day) or placebo. Cognitive functions were evaluated by neuropsychological tests prior to and following the intervention. The per-protocol set analysis (n = 76) revealed no significant differences between the exercise and the control groups in changes of neuropsychological tests. Subgroup analysis for participants with low skeletal muscle mass index (SMI) corresponding to sarcopenia cut-off value showed changes in selective attention, while working memory in the exercise with LCPUFA group was better than in the control group. These findings suggest that exercise with LCPUFA supplementation potentially improves attention and working memory in the elderly with low SMI.

## Introduction

Dementia is a severe social problem and taking measures to prevent it is important. Age-related cognitive decline is a substantial predicament regarding elderly people. It is widely recognized that exercise is one of the most reliable protective factors against cognitive decline. The World Health Organization (WHO) recommends that elderly people should exercise to reduce cognitive decline’s risk^1^. Moreover, several epidemiological studies showed exercise’s positive effects on the elderlies' cognitive functions^2,3^. WHO recommends 150 min/week and more moderate exercise for reducing the risk of cognitive decline^1^.

Apart from that, appropriate nutrients through healthy diets are also important in maintaining cognitive functions. The correlation between age-related cognitive decline and long-chain polyunsaturated fatty acids (LCPUFA), mainly included in fish, egg, and meat has been studied. LCPUFA, such as docosahexaenoic acid (DHA) and arachidonic acid (ARA), are major components in brain phospholipids. Although LCPUFA decreased with age in brain^4–6^, supplementation with these fatty acids could recover its levels^6^. Therefore, such supplementation is expected to ameliorate the age-related cognitive decline associated with depleted LCPUFA levels. Moreover, several clinical trials reported DHA's, eicosapentaenoic acid's (EPA), and ARA's supplementation on cognitive function efficacy improvement in the elderly^7–9^.

Recently, multifactorial lifestyle intervention is thought to be more effective than single factor intervention on improvement of cognitive function. The Finnish Geriatric Intervention Study to Prevent Cognitive Impairment and Disability (FINGER) study showed that the 2-year multi-domain intervention (moderate-intensity exercise, nutritional intervention, cognitive training, and vascular monitoring) reduced the risk of cognitive decline by 31% in non-demented elderly^10^. Referred to this study, the possibility was arisen that combining exercise with LCPUFA would be more beneficial to prevent cognitive decline than exercise alone. Regarding multifactorial intervention studies that included exercise and LCPUFA, the Multidomain Alzheimer Preventive Trial (MAPT) study showed that a 3-year multifactorial intervention by exercise, DHA/EPA supplementation, and cognitive training did not significantly affect cognitive decline^11^. Then, the subgroup analysis in MAPT study revealed the multifactorial intervention's tendency on improving attention in prefrail elderlies with memory complaints^12^. This finding potentially posits that the multifactorial lifestyle intervention including exercise and nutrition could provide additional beneficial effects on the elderly who are frail or sarcopenia prone. This could be partially supported by previous studies that frailty or sarcopenia was an important risk factor for age-related cognitive decline^13,14^. Therefore, we hypothesised that the combination of exercise and LCPUFA intake would be more effective than exercise alone on improving cognitive function in non-demented elderly, and the efficacy could be larger in those with frailty or sarcopenia tendency.

This study attempted to evaluate the effects of combining exercise with LCPUFA on cognitive function in non-demented elderly participants. We conducted a 24-week randomised control pilot trial to investigate the effects of moderate-intensity exercise (150 min/week) with LCPUFA (DHA 300 mg, EPA 100 mg and ARA 120 mg/day) supplementation combination on cognitive function in the non-demented elderly Japanese with cognitive decline complaint. Further subgroup analysis by low skeletal muscle mass index (SMI) corresponding to sarcopenia cut-off value were also performed to evaluate whether the combined intervention in this study had an added effect in elderly participants with sarcopenia tendency.

## Results

### Participants flow and baseline characteristic

**Figure 1:**
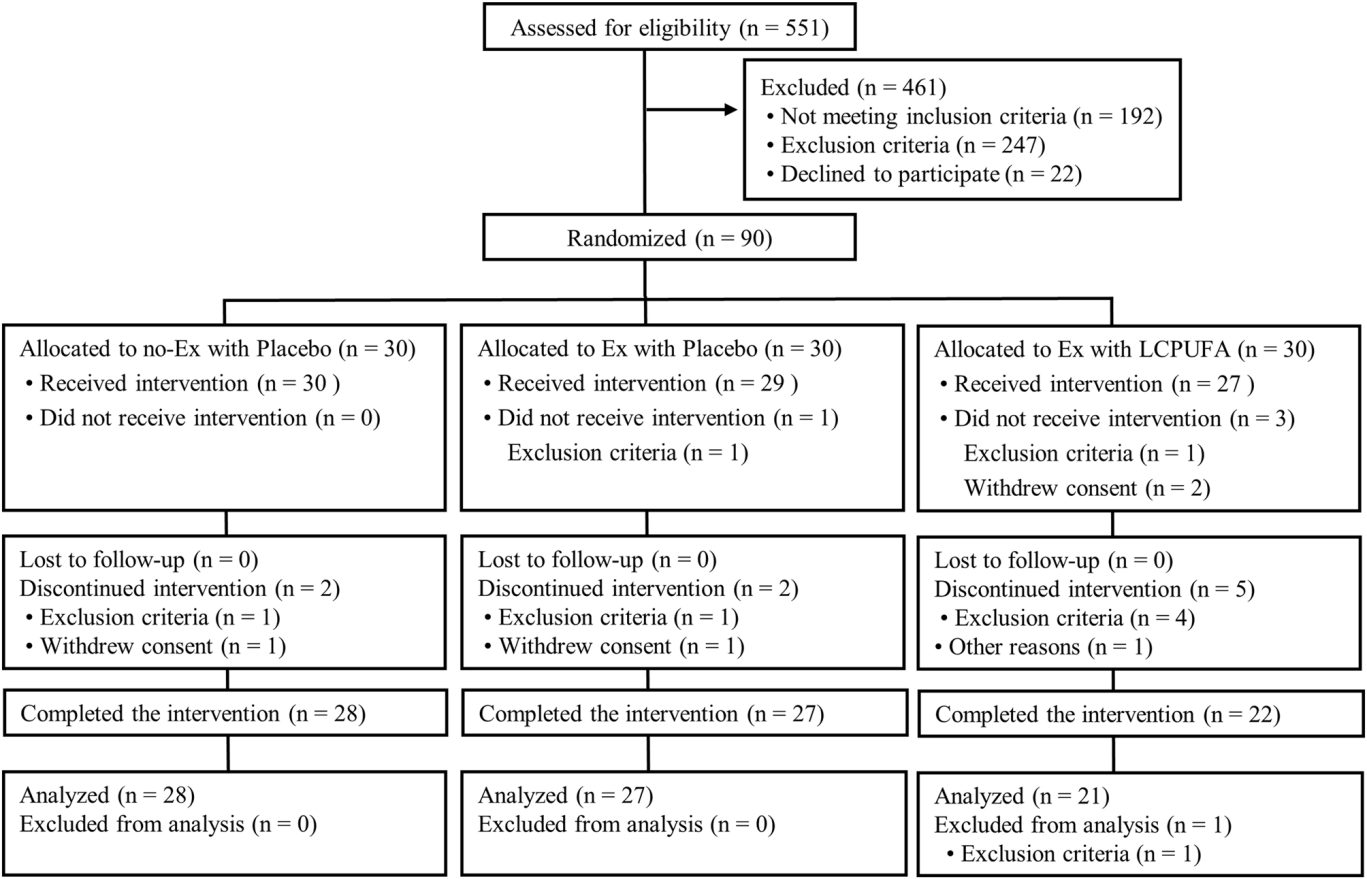
The CONSORT flowchart of this study.

The participant flow diagram is shown in Fig. 1. We screened a total of 551 participants; 90 of which were enrolled and randomly allocated to the groups (n = 30 in each group). Four participants (exercise with placebo, n = 1; exercise with LCPUFA, n = 3) were found to meet exclusion criteria for the entry or withdrew consent prior to the interventions. Eighty-six participants (no exercise with placebo, n = 30; exercise with placebo, n = 29; exercise with LCPUFA, n = 27) began the interventions and were involved in safety assessment (the full analysis set population). Nine participants discontinued the intervention for the following reasons: eventual discovery of entry exclusion criteria (n = 6), withdrawn consent (n = 2), and other reason (n = 1). Seventy-seven participants completed the 24-week intervention period, and 76 per-protocol set (PPS) population (no exercise with placebo, n = 28; exercise with placebo, n = 27; exercise with LCPUFA, n = 21) was used for efficacy assessment. One participant was excluded from the analysis due to a discovery to meet exclusion criteria for the entry. Here, only the PPS population results are shown because participants who completed the intervention (n = 77) were almost the same as the PPS population (n = 76). Mean compliances for the resistance and aerobic training program were 95.3 ± 1.0% and 92.8 ± 1.8% in the exercise with placebo, and 93.3 ± 1.3% and 94.9 ± 1.8% in the exercise with LCPUFA group, respectively. No significant differences in these compliances between the groups (resistance training, *p* = 0.199; aerobic training, *p* = 0.416) occurred. The experimental period's mean capsule intake was 98.8 ± 0.4% in the no exercise with placebo, 99.4 ± 0.2% in the exercise with placebo, and 99.1 ± 0.2% in the exercise with LCPUFA group. No significant difference in the capsule intake among the groups was observed (*p* = 0.357). Baseline characteristics are shown in Table 1. Age, sex, body mass index (BMI), education, cognitive function; the Japanese version of the Montreal Cognitive Assessment (MoCA-J) and Wechsler Memory Scale Revised logical memory (WMS-R LM II) scores, LCPUFA (DHA, EPA, and ARA) composition in plasma phospholipids, SMI, and physical activity (METs and steps) were matched among the groups.

**Table 1:** Baseline characteristics of the participants.

	no Ex + placebo	Ex + placebo	Ex + LCPUFA	*p*
	(n = 28)	(n = 27)	(n = 21)	
Age (year)^a^	67.4 ± 1.0	67.8 ± 0.8	67.1 ± 1.1	0.895
Sex (M/F)^b^	10/18	9/18	10/11	0.633
BMI (kg/m^2^)^a^	22.4 ± 0.5	22.7 ± 0.6	22.7 ± 0.6	0.906
Education (year)^a^	13.3 ± 0.4	13.7 ± 0.4	13.2 ± 0.6	0.777
MoCA-J^a^	22.2 ± 0.6	23.5 ± 0.5	23.5 ± 0.7	0.207
WMS-R LM II^a^	13.1 ± 0.8	13.4 ± 1.1	13.4 ± 1.1	0.972
LCPUFA in plasma PL				
ARA (%)^a^	10.0 ± 0.3	9.5 ± 0.3	9.7 ± 0.4	0.488
EPA (%)^a^	2.1 ± 0.2	2.2 ± 0.2	2.1 ± 0.3	0.938
DHA (%)^a^	7.6 ± 0.3	7.6 ± 0.3	7.1 ± 0.3	0.447
SMI (kg/m^2^)^a^	6.4 ± 0.2	6.2 ± 0.2	6.5 ± 0.2	0.701
Physical activity (METs/day)^a^	0.9 ± 0.0	0.9 ± 0.0	0.9 ± 0.0	0.800
Step (/day)^a^	6,716 ± 621	5,935 ± 419	7,807 ± 794	0.115

Mean ± SE. There was no significant difference among the groups in baseline data (^a^ANOVA, ^b^chi-square test). Ex, exercise; BMI, body mass index; MoCA-J, Montreal Cognitive Assessment Japanese version; WMS-R LM II, Wechsler memory scale-revised logical memory II; LCPUFA, long-chain polyunsaturated fatty acid; PL, phospholipids; ARA, arachidonic acid; EPA, eicosapentaenoic acid; DHA, docosahexaenoic acid; SMI, skeletal muscle mass index.

### Muscle mass and physical activity in PPS analysis

Muscle mass and daily steps are shown in Table 2. There were no significant differences in these aspects at baseline among the groups. Although muscle mass in the no exercise with placebo group was decreased by 0.5 kg, both groups performed muscle-maintenance exercises; however, changes in muscle mass were non-significant. Steps in the exercise with placebo group and with LCPUFA were increased significantly by 1,264 (*p* < 0.001 vs. baseline) and 780 steps/day (*p* = 0.041 vs. baseline), respectively. Changes in steps were significantly larger in the exercise with placebo or with LCPUFA comparing to the no exercise with placebo group (*p* < 0.001 and *p* = 0.039, respectively).

**Table 2:** Muscle mass, physical activity and fatty acid composition in plasma phospholipid in the groups during the intervention.

	Group	Baseline	24 weeks	Δ
Muscle mass (kg)	no Ex + placebo	37.8 ± 1.5	37.4 ± 1.5	− 0.5 ± 0.3
	Ex + placebo	36.2 ± 1.4	36.3 ± 1.4	0.2 ± 0.2
	Ex + LCPUFA	38.3 ± 1.7	38.3 ± 1.7	0.0 ± 0.1
Step (/day)	no Ex + placebo	6,716 ± 621	6,393 ± 563	− 322 ± 301
	Ex + placebo	5,935 ± 419	7,198 ± 383**	1,264 ± 311^##^
	Ex + LCPUFA	7,807 ± 794	8,587 ± 642*	780 ± 358^#^
FA composition in plasma PL				
PA (%)	no Ex + placebo	27.1 ± 0.2	26.7 ± 0.2*	− 0.4 ± 0.2
	Ex + placebo	26.9 ± 0.2	26.5 ± 0.2*	− 0.4 ± 0.2
	Ex + LCPUFA	27.0 ± 0.2	26.8 ± 0.3	− 0.3 ± 0.2
SA (%)	no Ex + placebo	14.4 ± 0.2	14.6 ± 0.2	0.2 ± 0.2
	Ex + placebo	14.7 ± 0.1	14.6 ± 0.2	− 0.1 ± 0.1
	Ex + LCPUFA	14.1 ± 0.3	14.4 ± 0.3	0.4 ± 0.2
OA (%)	no Ex + placebo	9.2 ± 0.2	9.1 ± 0.2	− 0.1 ± 0.2
	Ex + placebo	9.4 ± 0.2	9.0 ± 0.2*	− 0.5 ± 0.2
	Ex + LCPUFA	9.6 ± 0.2	8.9 ± 0.1**	− 0.7 ± 0.2
LA (%)	no Ex + placebo	19.9 ± 0.4	19.1 ± 0.5	− 0.7 ± 0.4
	Ex + placebo	19.9 ± 0.6	19.7 ± 0.5	− 0.1 ± 0.5
	Ex + LCPUFA	20.7 ± 0.6	18.9 ± 0.5**	− 1.8 ± 0.3
ARA (%)	no Ex + placebo	10.0 ± 0.3	9.8 ± 0.3	− 0.2 ± 0.2
	Ex + placebo	9.5 ± 0.3	9.7 ± 0.4	0.3 ± 0.3
	Ex + LCPUFA	9.7 ± 0.4	10.6 ± 0.3**	0.9 ± 0.2^##^
EPA (%)	no Ex + placebo	2.1 ± 0.2	2.7 ± 0.4	0.6 ± 0.4
	Ex + placebo	2.2 ± 0.2	2.7 ± 0.3	0.5 ± 0.3
	Ex + LCPUFA	2.1 ± 0.3	2.4 ± 0.2	0.3 ± 0.3
DHA (%)	no Ex + placebo	7.6 ± 0.3	7.4 ± 0.3	− 0.1 ± 0.2
	Ex + placebo	7.6 ± 0.3	7.6 ± 0.4	0.0 ± 0.2
	Ex + LCPUFA	7.1 ± 0.3	8.3 ± 0.2**	1.3 ± 0.2^##^
EPA/ARA	no Ex + placebo	0.22 ± 0.02	0.28 ± 0.05	0.07 ± 0.04
	Ex + placebo	0.25 ± 0.03	0.30 ± 0.04	0.05 ± 0.04
	Ex + LCPUFA	0.23 ± 0.04	0.23 ± 0.02	0.01 ± 0.03

Mean ± SE. no Ex + placebo (n = 28), Ex + placebo (n = 27) and Ex + LCPUFA (n = 21) groups. There was no significant difference among the groups for each fatty acid, muscle mass and step at baseline (one-way ANOVA). ^#^*p* < 0.05 and ^##^*p* < 0.01 vs. the no EX + placebo group (Dunnett's). *p < 0.05 and **p < 0.01 versus baseline (paired *t*-test). Ex, exercise; LCPUFA, long-chain polyunsaturated fatty acids; FA, fatty acid; PL, phospholipid; PA, palmitic acid; SA, stearic acid; OA, oleic acid; LA, linoleic acid; ARA, arachidonic acid; EPA, eicosapentaenoic acid; DHA, docosahexaenoic acid; EPA/ARA, ratio of EPA and ARA.

### Fatty acid compositions in plasma and dietary assessment in PPS analysis

Fatty acid compositions in plasma phospholipids are shown in Table 2. The DHA, EPA, and ARA content at baseline was not different among the groups. DHA and ARA compositions in the exercise with LCPUFA group at 24 weeks were increased significantly by 1.3% (*p* < 0.001 vs. baseline) and 0.9% (*p* = 0.001 vs. baseline), respectively. The DHA and ARA content in the no exercise and the exercise with placebo group remained unchanged during a period of intervention. Changes in the DHA and ARA content differed significantly between groups (*p* < 0.001 and *p* = 0.004 vs. no exercise with placebo, respectively). In terms of the EPA content, no significant differences were observed among the groups or between before and after the intervention. Fatty acid intakes from daily diets are shown in Supplementary Table S2. No significant differences were found in DHA, EPA, and ARA intake between groups or before and after supplementation. Moreover, intake changes of these fatty acids did not differ between groups. Regarding α-linolenic acid and linoleic acid, which are precursors of DHA/EPA and ARA, no significant differences were found among the groups or between before and after the intervention, while these changes did not differ between groups.

### Cognitive functions in PPS analysis

Table 3 shows the neuropsychological tests scores for attention, working memory, executive function, and episodic memory in the groups. There were significant differences in Stroop Colour-Word (CW) step 1 (*p* = 0.044) and step 3 (*p* = 0.014) at baseline among the groups. No significant differences were observed in other tests at baseline among the groups. In the exercise with placebo group, the scores of Stroop CW step 1, 2, Trail Making Test (TMT) -B, Digit Span, WMS-R LM I and II were significantly improved after the intervention for 24 weeks (vs. baseline). The significant improvement of WMS-R LM II score was observed in the exercise with LCPUFA group after the same period of intervention (vs. baseline). Changes in the scores of neuropsychological tests adjusted by individual baseline scores (Δ adjusted) did not differ between the exercise with placebo and the no exercise with placebo group; however, several scores in attention (Stroop CW step 1, + 3.3; TMT-A, − 3.7 s; TMT-B, − 7.1 s) were larger in the exercise with placebo than the no exercise with placebo group (Stroop CW step 1, + 1.0; TMT-A, − 1.0 s; TMT-B, − 0.3 s) and those effect sizes were small. Regarding the exercise with LCPUFA group, there were no significant differences in changes (Δ adjusted) of neuropsychological tests comparing the no exercise with the placebo group; however, a similar trend of the effect in the exercise with placebo group was observed. Scores in attention (Stroop CW step 1, + 3.7; TMT-B, − 5.1 s) and working memory (Digit span, + 1.3) were larger than the no exercise with placebo group (Digit span, + 0.7), and those effect sizes were small.

**Table 3:** Neuropsychological tests in the groups during the intervention.

	Group	Baseline	24 weeks	Change (Δ)	*r*	Δ adjusted	*r*
**Attention**							
Selective							
Stroop CW step 1	no Ex + placebo	52.9 ± 1.7	53.1 ± 1.9	0.2 ± 1.1	–	1.0 ± 1.5	–
	Ex + placebo	51.3 ± 1.7	54.3 ± 1.7**	3.1 ± 0.9	0.25	3.3 ± 1.5	0.24
	Ex + LCPUFA	45.8 ± 2.7	50.8 ± 2.7	5.0 ± 2.7	0.25	3.7 ± 1.7	0.13
Stroop CW step 3	no Ex + placebo	35.4 ± 1.2	36.0 ± 1.2	0.6 ± 0.5	–	1.1 ± 1.0	–
	Ex + placebo	35.1 ± 1.4	35.7 ± 1.4	0.6 ± 0.7	0.01	0.9 ± 1.0	0.01
	Ex + LCPUFA	29.8 ± 1.7	33.2 ± 1.9	3.4 ± 2.1	0.21	2.3 ± 1.2	0.07
Selective/divided							
TMT-A	no Ex + placebo	32.3 ± 2.3	32.6 ± 2.1	0.2 ± 2.1	–	− -1.0 ± 1.9	–
	Ex + placebo	32.2 ± 1.6	29.9 ± 1.6	− 2.4 ± 1.2	0.15	− 3.7 ± 2.0	0.17
	Ex + LCPUFA	38.8 ± 4.6	32.2 ± 2.9	− 6.6 ± 5.1	0.20	− 3.2 ± 2.3	0.08
Divided							
TMT-B	no Ex + placebo	74.9 ± 4.7	75.1 ± 4.8	0.2 ± 4.4	-	− 0.3 ± 4.1	–
	Ex + placebo	74.3 ± 5.1	67.9 ± 3.6*	− 6.4 ± 2.6	0.17	− 7.1 ± 4.2	0.21
	Ex + LCPUFA	80.5 ± 5.5	73.9 ± 8.2	− 6.6 ± 6.9	0.13	− 5.1 ± 4.8	0.10
**Working memory**							
Digit span	no Ex + placebo	12.7 ± 0.7	13.3 ± 0.8	0.6 ± 0.5	-	0.7 ± 0.5	–
	Ex + placebo	11.1 ± 0.6	12.0 ± 0.7*	0.9 ± 0.4	0.07	0.7 ± 0.5	0.05
	Ex + LCPUFA	11.9 ± 1.0	13.2 ± 1.0	1.3 ± 0.8	0.12	1.3 ± 0.6	0.10
**Executive function**							
Inhibitory control							
Stroop CW step 2	no Ex + placebo	43.6 ± 1.8	45.3 ± 1.9	1.7 ± 1.2	–	2.2 ± 1.5	–
	Ex + placebo	44.0 ± 1.6	47.7 ± 1.7**	3.6 ± 1.3	0.15	4.3 ± 1.5	0.17
	Ex + LCPUFA	38.4 ± 2.8	43.0 ± 2.4	4.6 ± 2.8	0.15	3.1 ± 1.7	0.04
Stroop CW step 4	no Ex + placebo	27.5 ± 2.1	29.5 ± 2.0	2.0 ± 1.4	–	2.1 ± 1.4	–
	Ex + placebo	28.3 ± 2.2	30.4 ± 2.0	2.1 ± 1.4	0.01	2.4 ± 1.4	0.02
	Ex + LCPUFA	25.8 ± 2.4	28.7 ± 2.0	2.9 ± 2.2	0.05	2.4 ± 1.6	0.01
Cognitive flexibility							
KWCST CA	no Ex + placebo	3.4 ± 0.4	3.2 ± 0.4	− 0.2 ± 0.3	–	− 0.1 ± 0.3	–
	Ex + placebo	3.0 ± 0.4	3.2 ± 0.4	0.3 ± 0.4	0.12	0.2 ± 0.3	0.09
	Ex + LCPUFA	3.3 ± 0.5	3.5 ± 0.4	0.1 ± 0.4	0.10	0.2 ± 0.4	0.11
Language flexibility							
Verbal fluency	no Ex + placebo	70.6 ± 2.3	72.5 ± 2.7	1.9 ± 1.8	–	1.6 ± 2.0	–
	Ex + placebo	70.9 ± 3.3	73.3 ± 3.2	2.4 ± 2.2	0.02	2.1 ± 2.0	0.03
	Ex + LCPUFA	76.0 ± 4.3	79.5 ± 4.4	3.6 ± 2.5	0.08	4.3 ± 2.3	0.12
**Episodic memory**							
Verbal immediate							
WMS-R LM I	no Ex + placebo	19.1 ± 1.0	21.1 ± 1.1*	2.0 ± 0.9	–	2.0 ± 1.0	–
	Ex + placebo	19.0 ± 1.0	21.0 ± 0.8*	2.1 ± 0.9	0.01	2.0 ± 1.1	0.00
	Ex + LCPUFA	19.0 ± 1.3	22.6 ± 2.0	3.6 ± 1.8	0.12	3.6 ± 1.2	0.13
Verbal delayed							
WMS-R LM II	no Ex + placebo	13.1 ± 0.8	17.3 ± 1.0**	4.2 ± 0.8	–	4.1 ± 1.0	–
	Ex + placebo	13.4 ± 1.1	16.3 ± 1.0**	3.0 ± 0.9	0.14	3.0 ± 1.1	0.14
	Ex + LCPUFA	13.4 ± 1.1	18.0 ± 2.1*	4.6 ± 1.7	0.03	4.6 ± 1.2	0.04
Visual delayed							
ROCFT recall	no Ex + placebo	14.8 ± 0.9	17.4 ± 1.1*	2.6 ± 1.2	–	2.4 ± 1.0	–
	Ex + placebo	14.0 ± 1.5	15.1 ± 1.2	1.2 ± 1.1	0.12	0.7 ± 1.0	0.18
	Ex + LCPUFA	17.6 ± 1.4	17.0 ± 1.5	− 0.6 ± 1.0	0.28	0.3 ± 1.1	0.21

Mean ± SE. no Ex + placebo (n = 28), Ex + placebo (n = 27) and Ex + LCPUFA (n = 21) groups.

There were significant differences in Stroop CW step 1 (*p* = 0.044) and step 3 (*p* = 0.014) at baseline among the groups. (one-way ANOVA). *p < 0.05 and **p < 0.01 vs. baseline (paired *t*-test). There was no significant difference in change (Δ) and change adjusted by baseline (Δ adjusted) between the either Ex groups and the no Ex + placebo (Dunnett's). Ex, exercise; LCPUFA, long-chain polyunsaturated fatty acids; Stroop CW, Stroop Colour-Word; TMT, Trail making test; KWCST CA, Wisconsin card sorting test of Keio version category achieved; WMS-R LM I/II, Wechsler memory scale revised logical memory I/II; ROCFT, Rey–Osterrieth complex figure test. Effect size is expressed as *r.*

### Subgroup analysis by low SMI

**Figure 2:**
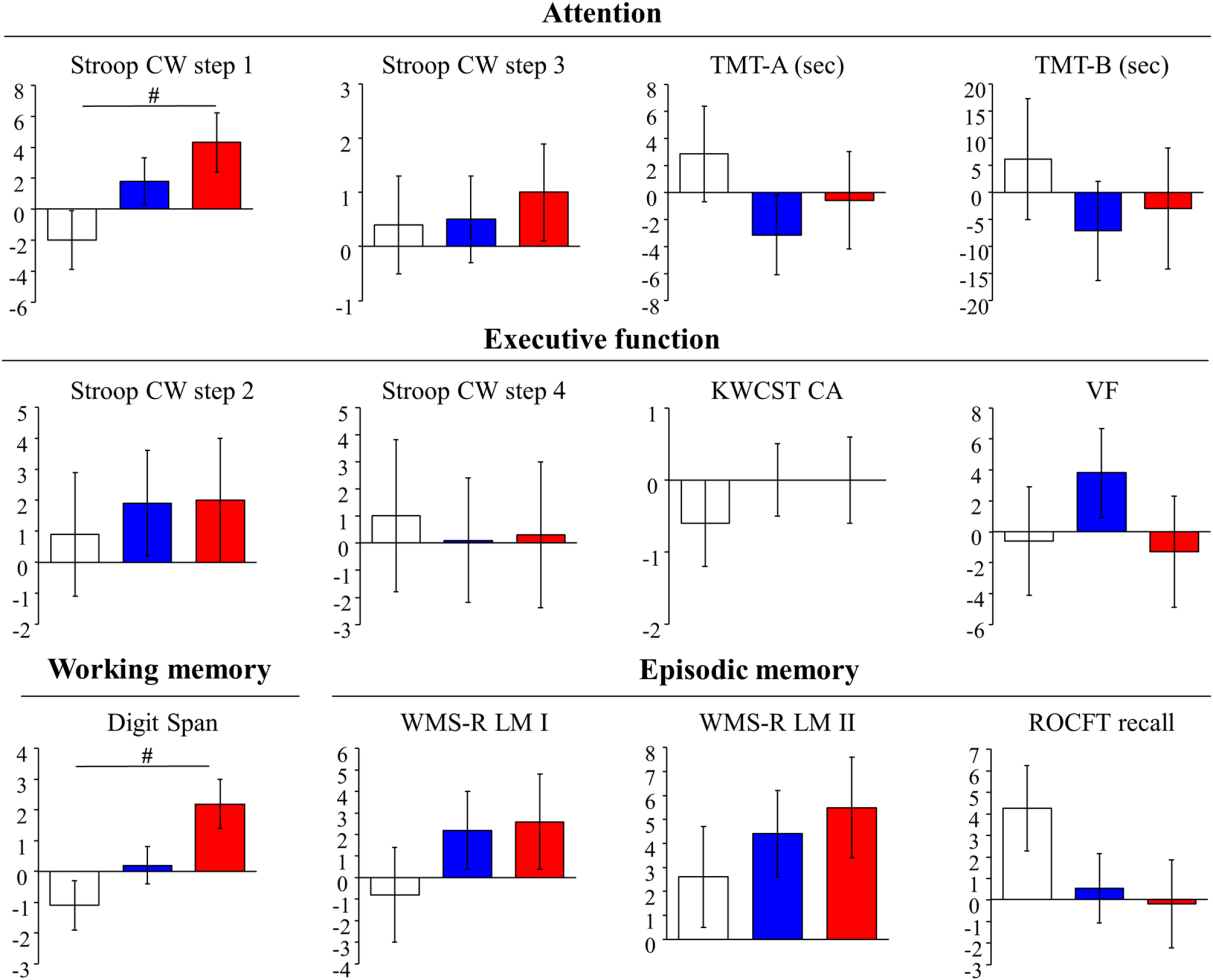
Changes in neuropsychological tests adjusted by baseline scores in the groups during the intervention in the subgroup with low SMI. Mean ± SE. White column, no Ex with placebo (n = 8); blue column, Ex with placebo (n = 12); red column, the Ex with LCPUFA (n = 8). #*p* < 0.05 vs. the no Ex with placebo group (Dunnett's). Stroop CW, Stroop Colour-Word; TMT, Trail making test; KWCST CA, Wisconsin card sorting test of Keio version category achieved; VF, Verbal fluency; WMS-R LM I/II, Wechsler memory scale revised logical memory I/II; ROCFT, Rey–Osterrieth complex figure test; SMI, skeletal muscle mass index.

The subgroup analysis by low SMI corresponding to the sarcopenia cut-off value was conducted and twenty-eight participants (no exercise with placebo, n = 8; exercise with placebo, n = 12; exercise with LCPUFA, n = 8) were analysed. The baseline characteristics are shown in Supplementary Table S3, and there were no significant differences in any factor among the groups. The effects of the interventions on muscle mass, steps, and LCPUFA content in plasma phospholipids in the subgroup analysis were similar to those in the PPS analysis (Supplementary Table S4). Scores of neuropsychological tests for cognitive domains before and after the intervention were shown in Supplementary Table S5. No significant differences in scores at baseline were observed among the groups. Figure 2 shows changes (Δ adjusted) in scores of neuropsychological tests. There were no significant differences in changes of neuropsychological tests between the exercise with placebo and the no exercise with placebo group; although, changes of several scores in attention (Stroop CW step 1, TMT-A and B) in the exercise with placebo group were larger than those in the no exercise with placebo group as those effect sizes were small or middle. Changes (Δ adjusted) of selective attention (Stroop CW step 1) and working memory (Digit Span) were + 4.3 (*p* = 0.049) and + 2.2 (*p* = 0.013) in the exercise with LCPUFA group and were significantly larger than those (− 2.0 and − 1.1) in the no exercise with placebo group as those effect sizes were large (*r* = 0.52 and 0.59). No significant differences in changes of other neuropsychological tests between both groups were observed. Changes of attentional scores (Stroop CW step 3, TMT-A and B) in the exercise with LCPUFA group were larger than those in the no exercise with placebo group, and those effect sizes were small.

### Safety

The safety assessment was performed with the full analysis set population (n = 86). No side effects due to the LCPUFA-containing supplements were observed. There were no severe adverse events and no significant difference (*p* = 0.480) in the incidence of adverse events between the no exercise with placebo (50.0%), the exercise with placebo (65.5%), and the exercise with LCPUFA groups (59.3%).

## Discussion

In this study, we conducted a randomised, single-masked, placebo-controlled pilot trial for 24 weeks to evaluate effects of moderate-intensity exercise (150 min/week) with LCPUFA (DHA 300 mg, EPA 100 mg and ARA 120 mg/day) supplementation on cognitive functions in non-demented elderly people with cognitive decline complaints. In PPS analysis (n = 76), neither the exercise nor the exercise with LCPUFA supplementation reached significant effects on cognitive functions. However, significant improvements of exercise with LCPUFA supplementation on selective attention and working memory were observed in participants with low SMI in subgroup analysis (n = 28). This is a first report suggesting that exercise with nutritional supplementation comprised LCPUFA could potentially improve attention and working memory in the elderly with tendency to sarcopenia.

In PPS analysis, we confirmed that the participants in this study had slightly age-related cognitive decline because scores in neuropsychological tests, such as Stroop CW step1 (50.3), Digit Span (11.9) WMS-R LM II (13.3) and Wisconsin Card Sorting Test of Keio version (KWCST) (3.2) at baseline were lower comparing to previous reports (52.2^15^, 12.8^16^, 15.3^16^ and 4.2^17^ in typical age-matched elderly people. Then, there were no marked differences in daily steps reflecting physical activity between this study (men 7,700 and women 6,200 steps) and the typical elderly individuals (men 6,700 and women 5,800 steps)^18^. In terms of LCPUFA, DHA (7.4%), EPA (2.1%) and ARA (9.7%) compositions in plasma PL and dietary DHA (570 mg/day), EPA (340 mg/day) and ARA (180 mg/day) intakes were in a range of previous studies in the elderly Japanese^9,19–23^. This suggests that the participants in this study were on-target and general population in the elderly Japanese.

A number of RCT reported that exercise for 1–12 months improved cognitive functions such as executive function and attention as evaluated by Stroop CW^24–27^. Conversely, we found no significant effect of exercise regarding 150 min/week moderate-intensity resistance and aerobic training program on cognitive functions under this study condition; although, we observed the attention-improving tendency as measured by Stroop CW, TMT. Van de Rest also reported that the exercise for the 24 weeks similar program to the present study showed no significant effects on Stroop CW^28^. One of the reasons our exercise program did not reach to the significant effect could be insufficient of specimens and also of intensity or duration. The exercise program of 150 min/day with moderate intensity was considered as the minimum requirement of WHO recommendation to reduce the risk of cognitive decline in the elderly^1^. Therefore, higher intensity or longer time exercise could have more clear effects on cognitive functions, such as attention, in the non-demented elderly with cognitive decline complaints.

The primary purpose of this pilot study was to evaluate whether combination of exercise with LCPUFA supplementation was more effective comparing to only exercise on cognitive functions in the elderly. In the PPS analysis, we did not find clear effects on any cognitive domains; although, changes in some scores, such as attention and working memory as evaluated by Stroop CW step 1 and Digit Span in the exercise with LCPUFA groups had a slight increasing tendency in contrast to the exercise group. Further studies are necessary to clarify the effect of combining exercise and LCPUFA supplementation on cognitive decline in an RCT with larger sample size. Then, the SMI subgroup analysis reflecting muscle mass was performed as frailty or sarcopenia has recently been highlighted as a remarkable risk factor for age-related cognitive decline^13,14,29^. The frail or sarcopenic elderly are considered to be a vulnerable population to cognitive decline because of decreased protective factors, such as neurotrophic factor and growth hormone, and increased risk factors, such as inflammation, insulin resistance, and oxidative stress^13,14,29^. It is well understood that frailty or sarcopenia are correlated with age-related muscle decrease and also reported that age-related muscle loss tended to be accelerated in over 60 years old^30^. In the subgroup analysis with SMI below cut-off value of sarcopenia, the combination of exercise with LCPUFA supplementation improved selective attention and working memory; although, only exercise did not reach to significant effects. This finding was supported by a similar trend that was also found in the additional subgroup analysis by SMI below the median in this study (data not shown). In addition, the data from our previous study also corresponded to our findings. The subgroup analysis in MAPT study revealed a multifactorial intervention including exercise, DHA/EPA supplementation and cognitive training tended to improve attention in elderly with prefrail condition; although, no significant effect was observed^12^. In terms of cognitive domains affected by exercise with LCPUFA, attention and working memory could be reasonable. It was showed that the exercise might have attention-improving potential rather than other cognitive domain under this study condition. It was also reported that LCPUFA supplementation with similar dose in the present study had a beneficial effect on the P300 latency^9^ reflecting attention and working memory^31,32^. Here, the differences in changes between the exercise with LCPUFA and the control group in the scores were 6.3 (Stroop CW step1) and 3.3 (Digit Span) and were considered as physiologically meaningful due to these scores being decreased with age by approximately 0.8/year and 0.1/year in typical elderly Japanese, respectively^15,16^. Taken together, these data suggest that moderate-intensity exercise with LCPUFA supplementation in a range of general daily intake could have improved potential than only exercise to improve attention and working memory in the elderly with low SMI.

In terms of LCPUFA's contribution to the efficacy of the combination with the exercise, our results suggest that the efficacy might be caused by DHA and ARA mainly. It was reported that DHA and ARA play important roles on brain function, such as synaptic plasticity, neurogenesis, and membrane fluidity^6,33–36^, but these LCPUFA decreased with age in the brain^4–6^. Our results show that the combination of the exercise and the LCPUFA supplementation in this study increased blood DHA and ARA composition; although, exercise alone had little effect on LCPUFA. Those LCPUFA increases might also be observed in the brain as blood DHA and ARA compositions were correlated to those in the brain^6^. These data suggest that the effect of combining the exercise with the LCPUFA supplementation could arise from administering continuous exercise stimulation to the brain with the amelioration of DHA and ARA decrease.

Here, our data did not explain the specific mechanism for the efficacy of combining the exercise with the LCPUFA supplementation on the cognitive functions in the elderly with low SMI. However, previous studies can allow us to hypothesise that exercise and LCPUFA supplementation work cooperatively in the elderly with low SMI to improve cognitive functions with increased protective factors and decreased cognitive decline-related risk factors. The elderly with low SMI are considered to be more prone to frailty or sarcopenia. As mentioned above, it was reported that protective neurotrophic factor, growth hormone and risk factors, inflammation, insulin resistance, and oxidative stress, for cognitive decline were increased and decreased in the elderly with frail or sarcopenia^13,14^, respectively. Conversely, improvements of these factors by both exercise^13,37^ and LCPUFA were reported^38–40^. Further studies are necessary to evaluate the effects of the combination exercise and LCPUFA supplementation on protective and risk factors for cognitive decline in the elderly with low SMI.

Although various cognitive domains were evaluated in this study, only selective attention and working memory were significantly improved by the exercise with the LCPUFA supplementation in the elderly with low SMI. Presently, ruling out the possibility that other cognitive domains, such as episodic memory were affected by the intervention is impossible. For instance, practice effects are often observed in neuropsychological memory tests more than in the tests for attention. In fact, significant increases of WMS-R LM between before and after the intervention in the control group were observed in the PPS analysis (Table 3). Therefore, the intervention's effect on other cognitive domains should be evaluated by different types of neuropsychological tests.

There were three limitations to this study. First, it was designed as a pilot study, and the major finding in the elderly with low SMI was based on the analysis of a scarcely populated subgroup. Second, the isolated effect of the LCPUFA supplementation on cognitive function was not investigated in this study since the design was to evaluate the hypothesis that the combination of exercise and LCPUFA intake would be more effective than exercise alone on improving cognitive function. Finally, the precise mechanisms remained unclear. Confirmatory studies with a larger sample size are needed to clarify the efficacy although this is the first report evaluating the effect on cognitive function in the non-demented elderly of the combination of exercise with nutritional supplementation comprised LCPUFA. Conversely, this study has some strength. The participants' intervention compliance was high. The mean capsule intake and participation of the exercise program for the experimental period were 100% and > 90%, respectively in the PPS analysis population. In addition, the validity of this study's interventions was high. It was considered that exercise and LCPUFA supplementation were performed appropriately based on the study design. Muscle mass in both exercise groups tended to increase (0.0–0.2 kg) compared to the no exercise group (− 0.5 kg) but the difference was not significant. Then, daily steps were increased by 780–1,260 steps/day in both exercise groups. The amount of increase was in a reasonable range according to the expectations (1,200 steps/day) based on the study protocol. Significant increases in DHA (+ 1.3%) and ARA (+ 0.9%) contents in plasma PL were observed in the exercise with LCPUFA group. The amounts of increases in DHA and ARA contents are considered within a reasonable range and physiologically meaningful as the increase of DHA and ARA contents was shown to be 0.9% and 0.6%, respectively, by similar doses of LCPUFA supplementation that had the potential to improve cognitive function^9^.

Regarding safety, we observed no side effects of LCPUFA supplementation, while there was no significant difference in the incidence of adverse events among groups. Thus, the combination of the exercise and the LCPUFA supplementation in the present study is considered safe under the conditions described here.

In conclusion, we did not find a clear effect of exercise with LCPUFA supplementation combination on cognitive function in the non-demented elderly with cognitive decline complaints under this study condition. However, exercise with LCPUFA supplementation could potentially improve attention and working memory in the elderly with low SMI. Therefore, a combination of exercise and nutritional approach could be beneficial for age-related cognitive decline.

## Methods

### Study design

We designed a randomised, single-blind, placebo-controlled, parallel group intervention attempting to evaluate the effect of combination exercise with LCPUFA supplementation on cognitive function in non-demented elderly Japanese with cognitive decline complaints during the time between November 2017 and December 2018 at a medical facility in Kita-ku, Osaka, Japan. Participants were recruited in Osaka and its environs. We screened 551 participants while 90 were randomly allocated to the no exercise with placebo (as a control), or the exercise with placebo, or the exercise with LCPUFA groups. Participants were administered the intervention (exercise and/or supplementation) for 24 weeks between April and December 2018. Both MoCA-J^41^ and WMS-R LM II^16^ were used for screening as described below. Blood and urine were sampled following overnight fasting for safety assessment and fatty acid analysis during the screening period, baseline, and 24 weeks following the intervention. Neuropsychological tests were performed, and muscle mass, physical activity and dietary fatty acid intake were measured at baseline and 24 weeks following the intervention. A study diary and pedometer were distributed and collected during intervention period. This study was registered in the University Hospital Medical Information Network (UMIN) Clinical Trial Registry (UMIN000030065) on 21 November 2017. The Aisei Hospital Ueno Clinic Research Ethics Committee approved the study protocol (#171109-1), which conformed to the principles set forth in the Declaration of Helsinki. Written informed consent was obtained from all participants. This study was reported based on The Consolidated Standards of Reporting Trials (CONSORT) statement (https://www.consort-statement.org/).

### Participants

Japanese participants constituted aged 60–79 years not exercising regularly and complaining about their own age-related cognitive (such as memory) decline with lower memory scores (WMS-R LM II < 20) than the reference in Japanese middle-aged adults^16^. Exclusion criteria were: weak vision; colour blindness; hearing loss; a history of neurological disorder or serious disorders and clinically significant systemic diseases; postmenopausal syndrome or hormone therapy; problems receiving exercise interventions; gelatine or olive oil allergy; an irregular lifestyle; heavy drinker; heavy smoker; a history of measurement of neuropsychological testing a year prior to the entry; consumption of drugs or supplements that affect efficacy evaluation, such as lipid metabolism or muscle metabolism or brain function; and dementia or suspicion of dementia (MoCA-J score < 17). This criterion for MoCA-J corresponds to the Mini-Mental State Examination (MMSE) score < 24 which is the most widely used screening for dementia^42^, and was set according to the conversion table between MoCA and MMSE^43^.

### Interventions

#### Exercise

This study’s purpose was to evaluate the hypothesis that the combination of exercise with LCPUFA supplementation was more effective than only exercise regarding the improvement of cognitive function. Therefore, the exercise condition was set referring to the WHO's minimum requirement to reduce the risk of cognitive decline in the elderly^1^. The guideline prescribes that at least 150 min per week of moderate-intensity exercise (3–6 METs)^44^ was needed for the elderly, and muscle-strengthening training involving major muscle groups should be performed 2 or more days a week. Then, participants in the exercise groups performed 150 min of exercise per week comprised 30 min resistance training for 2 weekly days, and 30 min aerobic exercise for 3 days of the week, for a period of 24 weeks. Each exercise's intensity was set referring to the table of METs for physical activities^45^. Participants in the no exercise with placebo group did not receive any exercise programs during the intervention period.

### Resistance training

The resistance training program (48 workouts total) was conducted at the local gym in Kita-ku Osaka every 2–3 days. Safety and appropriateness training made one-to-one basis participant supervision necessary by the well-trained instructors throughout the program. It constituted 30 min of resistance training, with 10 min warm up and 10 min cool down. Resistance training mainly focused on the major muscle group comprising six free weight trainings (squat, bench press, rowing, side raise, calf raise and sit up). Although the participants' instruction was to carry out five sets with approximately 10 repetition maximum (RM) per set in squat, and three sets with same intensity in other five training regimes, they could learn appropriate forms of all trainings and gradually increased intensities during the first four weeks of the 24-week period. The attendance and number of sets completed were recorded in the logs for each participant of every class by the trainers. Compliance, expressed as the percentage of the total programs attended and completed appropriately, was calculated from these logs.

### Aerobic training

The walking program aerobic training (72 total) was performed during all days, except for the day of resistance training. The program was conducted within the area of residence of each participant. Participants were instructed to increase their strides and swing their arms with the intensity of 12–13 rate of perceived exertion (RPE) during this program. The program constituted 30 min of walking with a few min of warm up and cool down. The attendance and steps for the program were recorded in the pedometers for each participant. Compliance, as the percentage of the total programs attended and completed appropriately, was calculated from pedometer logs. Walking programs were monitored by logs of pedometers and participant's self-records. The instructors checked those logs and records and gave feedbacks to participants when they joined the resistance training program.

### Experimental supplement

Experimental supplements included purified olive oil and LCPUFA-containing oil, as per the previous study^9^. Fatty acid compositions of these supplements are shown in Supplementary Table S1. The exercise with LCPUFA group consumed 1,080 mg/day of LCPUFA-containing oil in 6 soft gelatine capsules, in which 300 mg of DHA, 100 mg of EPA, and 120 mg of ARA were included as free fatty acid equivalents. The dose of LCPUFA was designed to be in a range of that from normal daily diets in elderly Japanese^23,46^. The same amount of purified olive oil was administered in the no-exercise group with placebo and in the exercise with placebo group. Capsules of LCPUFA and placebo were same size and colour. Compliance of the capsule intake in each participant was checked by the study diary.

### Outcome assessments

The primary outcome in the present study was the change in the score of Stroop CW test. Secondary outcomes were the changes in other neuropsychological tests. To confirm the validity of the intervention, we assessed muscle mass, physical activity, LCPUFA content in blood phospholipids, and dietary LCPUFA intake. Safety was assessed based on the incidence of side effects and adverse events among groups throughout a period of 24-week intervention.

### Cognitive functions assessment

To evaluate the effects of the interventions on cognitive functions, we assessed a wide range of cognitive functions classified into four major domains (attention, working memory, executive functions, and episodic memory) using neuropsychological tests. Regarding attention, selective attentions were measured by Stroop CW test step 1, 3^15^ and TMT-A^47^, while divided attention was measured by TMT-A, B^47^. In Stroop CW step 1 and 3, we evaluated the number of correct items in 60 s. In TMT-A and B, we evaluated the time(s) needed to finish the task. Working memory was measured by Digit Span^16^. The score in Digit Span comprises the sum of the forward and backward scores, and ranges from 0 to 24. Regarding executive functions, inhibitory control, cognitive flexibility and language flexibility were measured by Stroop CW step 2, 4^15^, KWCST^48^ and Verbal Fluency^49^. In Stroop CW step 2 and 4, we evaluated the number of correct items in 60 s. The KWCST was administered using a computerized version (WCST-KFS)^50^, and evaluated the score of category achieved (CA), ranging from 0 to 6. In Verbal Fluency, the score comprises the sum of the letter (shi, i and re) and the category (animals, fruits and vehicles) scores. The participants were instructed to produce as many words as possible from each task in 60 s. Regarding episodic memory, we measured verbal immediate/delayed memory and visual delayed memory by WMS-R LM I/II^16^ and Rey-Osterrieth Complex Figure Test (ROCFT) recall^51^ , respectively. Scores in WMS-R LM and ROCFT recall range from 0 to 50 and from 0 to 36. WMS-R LM II was performed 30 min following WMS-R LM I completion. ROCFT recall was conducted 3 min after coping the complex figure. Lower scores in TMT-A, B and higher scores in other neuropsychological tests represent better cognitive functions.

### Muscle mass and physical activity measurements

We analysed the whole-body muscle mass (kg) by multi-frequency bioelectric impedance analysis (InBody720, Biospace, Seoul, Korea). Regarding subgroup analysis by low SMI corresponding to sarcopenia cut-off value, SMI (kg/m^2^) was calculated by appendicular skeletal muscle divided square of height. Asian Working Group for Sarcopenia (AWGS) reported that the values of < 7.0 and < 5.7 kg/m^2^ in men and women were used as cut off levels of the screening for sarcopenia in Asian adults^52^. Physical activities (steps and METs) were measured by pedometers (Active style Pro HJA-750C; Omron Healthcare, Kyoto, Japan) for 24 weeks. The daily average physical activities were evaluated by averages of seven-day activities prior to the intervention's commencement and completion.

### Fatty acid analysis

Blood samples were centrifuged at 2,200 × *g* for 5 min at 4 °C, and separated to plasma. Samples were stored at − 80 °C prior to fatty acid analysis. Lipids in plasma were extracted and purified by the method of Bligh and Dyer^53^. Then, phospholipid fraction was separated by thin-layer chromatography with hexane:ether = 7:3 and incubated with an additional internal standard (pentadecanoic acid) in methanolic HCl at 50 °C for 3 h for transmethylation of fatty acid residues. Fatty acid methyl esters were extracted with *n*-hexane and analysed by gas–liquid chromatography (Agilent 7890B, Agilent Technologies, Santa Clara, CA, USA) as described previously^9^. The composition of each fatty acid was expressed as a percentage of the total peak area of the identified fatty acids.

### Dietary assessment and study diary

We performed dietary assessment according to a previous study^9^. We estimated dietary intake including LCPUFA (DHA, EPA and ARA) and their precursor (α-linolenic and linoleic acid) using an ad hoc computer algorithm for the BDHQ based on the Standard Tables of Food Composition in Japan 2010. Participants were instructed to keep a record in the study diary throughout the study.

### Sample size

We found that selective attention/executive function evaluated by the Stroop CW test tends to be more affected by exercise in the non-demented elderly based on more than twenty previous RCTs. We selected the trial of which primary outcome was set to Stroop CW test in the elderly Japanese^24^ as a representative RCT with exercise including resistance and aerobic training. Additionally, it was reported that the same dose of LCPUFA (DHA, EPA and ARA) of the present study had the efficacy on the P300 latency which correlated to the score of Stroop CW test in the elderly Japanese^9^. Regarding exercise intensity and intervention period, the required sample size (90 participants) was calculated based on above two studies with a 30% dropout rate. Sixty-three participants (21 participants in each group) in the per-protocol analysis would have 80% power at a 5% level of significance to detect differences in Stroop CW test changes between the no exercise with placebo and the exercise with LCPUFA groups.

### Randomisation and allocation

Enrolled participants were randomly assigned in a 1:1:1 ratio based on dynamic allocation to achieve balance among the groups regarding age, sex, WMS-R LM II, ARA and DHA composition in plasma phospholipids by using a spread sheet program with RAND function of Microsoft Excel 2013. The randomisation procedure was performed by a person who was not involved in this study. Following, the randomisation codes for these participants and the codes for masked supplements were each held in sealed opaque envelopes by 2 different individuals who were uninvolved in this study. Information about these assignments was masked to researchers until all data were collected and analysed.

### Blinding

Although this study design was single blinded RCT, participants were blinded to the supplementation (the placebo or the LCPUFA) in the exercise groups. Researchers involved in the assessment of outcome measures were blinded to the randomisation assignment.

### Statistics

The main efficacy assessment was performed with the PPS analysis as defined by the statistical analysis plan. Further subgroup analysis by low muscle mass was conducted also according to the statistical analysis plan. The sarcopenia cut-off value of SMI was used for this subgroup analysis as described in the muscle mass measurement. Safety assessment was performed with the full analysis set. Baseline data among the groups were compared by one-way analysis of variance (ANOVA) for quantitative variables and by chi-square test for qualitative variables. A change from baseline to 24 weeks after the intervention in each group was compared by paired *t*-test. Comparisons of changes between either exercise groups and the no exercise with placebo group were performed by Dunnett’s test. In addition, Dunnett’s tests that adjusted by baseline cognitive scores as a covariate were also performed, as we found that scores for cognitive function strongly affected on neuropsychological score changes by our preliminary study. Effect sizes were also calculated based on point-biserial correlation coefficient and expressed as *r* (*r* ≥ 0.10 is regard as a small, ≥ 0.30 is a middle and ≥ 0.50 is a large effect). The incidence of side effects and adverse events among groups was compared by chi-square test. Statistical analysis was performed using SPSS statistics 23 and higher (IBM-Armonk, New York, USA) and SAS version 9.4 (SAS Institute, Inc., Cary, NC, USA). Data are shown as mean ± standard error (SE). All tests were two-sided, and an alpha-level of 0.05 was considered statistically significant.

## Supplementary information

Supplementary Information.

## Data Availability

All data that support the findings of this study are included in this published article (and its Supplementary Information files).
