# Supplementary material for: Effects of combining exercise with long-chain polyunsaturated fatty acid supplementation on cognitive function in the elderly: a randomised controlled trial
Source: Sci Rep. 2020 Jul 31;10:12906. doi: 10.1038/s41598-020-69560-4 (PMC7395090; doi:10.1038/s41598-020-69560-4)
Supplement: Supplementary file 1 — Supplementary Information. [file 41598_2020_69560_MOESM1_ESM.docx]

**Effects of combining exercise with long-chain polyunsaturated fatty acid supplementation on cognitive function in the elderly: A Randomised controlled trial**

**Hisanori Tokuda^1*^, Mika Ito^2^, Toshiaki Sueyasu^1^, Hideyuki Sasaki^1^, Satoshi Morita^1^, Yoshihisa Kaneda^1^, Tomohiro Rogi^1^, Sumio Kondo^3^, Motoki Kouzaki^4^, Takashi Tsukiura^5^ and Hiroshi Shibata^1^**

^1^Institute for Health Care Science, Suntory Wellness Ltd., Kyoto, Japan

^2^Safety Science Institute, Suntory MONOZUKURI Expert Ltd., Kyoto, Japan

^3^Medical Corporation Kenshokai, Fukushima Healthcare Center, Osaka, Japan,

^4^Laboratory of Neurophysiology, Graduate School of Human and Environmental Studies, Kyoto University, Kyoto, Japan

^5^Department of Cognitive and Behavioral Sciences, Graduate School of Human and Environmental Studies, Kyoto University, Kyoto, Japan

*Corresponding author:

Institute for Health Care Science, Suntory Wellness Ltd.,

8-1-1 Seikadai, Seika-cho, Soraku-gun, Kyoto 619-0284, Japan

Tel +81 50 3182 0662 Fax +81 774 98 6262

E-mail Hisanori_Tokuda@suntory.co.jp

**Supplement Table S1.** Fatty acid composition in experimental supplements

Placebo LCPUFA

(Purified olive oil) containing oil

FA (%) (%)

Palmitic acid 16:0 10.2 6.4

Stearic acid 18:0 3.5 3.3

Arachidic acid 20:0 0.4 0.5

Behenic acid 22:0 0.1 1.0

Lignoceric acid 24:0 - 2.2

Palmitoleic acid 16:1 0.8 2.2

Oleic acid 18:1 73.4 8.2

Eicosenoic acid 20:1 0.3 1.5

Docosenoic acid 22:1 - 1.2

Tetracosenoic acid 24:1 - 0.6

Linoleic acid 18:2n-6 9.0 2.9

Eicosadienoic acid 20:2n-6 - 0.3

Dihomo-γ-linolenic acid 20:3n-6 - 1.1

ARA 20:4n-6 - 12.6

Docosatetraenoic acid 22:4n-6 - 0.4

Docosapentaenoic acid 22:5n-6 - 1.5

α-Linolenic acid 18:3n-3 1.2 0.4

EPA 20:5n-3 - 10.3

Docosapentaenoic acid 22:5n-3 - 2.2

DHA 22:6n-3 - 30.9

Others 1.1 10.3

Total 100.0 100.0

FA, fatty acid; ARA, arachidonic acid; EPA, eicosapentaenoic acid; DHA, docosahexaenoic acid.

**Supplementary Table S2. Dietary assessment in the groups during the intervention**

Group Baseline 24 weeks Δ

Energy (kcal/day) no Ex + placebo 1,689 ± 94 1,671 ± 84 -18 ± 67

Ex + placebo 1,690 ± 92 1,606 ± 102 -84 ± 66

Ex + LCPUFA 1,833 ± 142 1,910 ± 137 77 ± 92

Carbohydrate (g/day) no Ex + placebo 232 ± 16 226 ± 13 -6 ± 11

Ex + placebo 229 ± 14 219 ± 15 -10 ± 11

Ex + LCPUFA 228 ± 15 246 ± 19 18 ± 17

Protein (g/day) no Ex + placebo 64 ± 4 64 ± 4 0 ± 3

Ex + placebo 68 ± 5 64 ± 6 -3 ± 3

Ex + LCPUFA 73 ± 7 72 ± 6 -1 ± 3

Fat (g/day) no Ex + placebo 51 ± 3 51 ± 3 1 ± 2

Ex + placebo 52 ± 3 50 ± 4 -2 ± 2

Ex + LCPUFA 61 ± 6 63 ± 5 2 ± 3

Fatty acid

LA (g/day) no Ex + placebo 10.0 ± 0.6 10.1 ± 0.7 0.1 ± 0.5

Ex + placebo 9.4 ± 0.7 9.3 ± 0.9 -0.1 ± 0.5

Ex + LCPUFA 11.6 ± 1.1 12.2 ± 1.0 0.7 ± 0.6

ARA (mg/day) no Ex + placebo 167 ± 12 161 ± 14 -6 ± 11

Ex + placebo 186 ± 16 166 ± 15 -20 ± 10

Ex + LCPUFA 199 ± 27 195 ± 25 -4 ± 10

ALA (g/day) no Ex + placebo 1.6 ± 0.1 1.6 ± 0.1 0.0 ± 0.1

Ex + placebo 1.5 ± 0.1 1.5 ± 0.1 0.0 ± 0.1

Ex + LCPUFA 1.9 ± 0.2 2.0 ± 0.2 0.1 ± 0.1

EPA (mg/day) no Ex + placebo 315 ± 38 310 ± 32 -5 ± 33

Ex + placebo 333 ± 45 338 ± 43 5 ± 38

Ex + LCPUFA 378 ± 56 320 ± 38 -58 ± 43

DHA (mg/day) no Ex + placebo 537 ± 59 522 ± 50 -15 ± 49

Ex + placebo 567 ± 71 562 ± 67 -5 ± 55

Ex + LCPUFA 645 ± 92 556 ± 67 -89 ± 65

Mean ± SE. no Ex + placebo (n = 27), Ex + placebo (n = 27) and Ex + LCPUFA (n = 21) groups. One participant’s data in the no Ex + placebo group was lacked in the dietary assessment. There was no significant difference among the groups for each fatty at baseline (one-way ANOVA). There was no significant difference between baseline and 24 weeks after the intervention in each group (paired *t*-test). There was no significant difference in change (Δ) between the either Ex groups and the no Ex + placebo (Dunnett's). Ex, exercise; LCPUFA, long-chain polyunsaturated fatty acids; LA, linoleic acid; ARA, arachidonic acid; ALA, a-linolenic acid; EPA, eicosapentaenoic acid; DHA, docosahexaenoic acid.

**Supplementary Table S3. Baseline characteristics of the participants in subgroup analysis by SMI < AWGS**

no Ex + placebo Ex + placebo Ex + LCPUFA

(n = 8) (n = 12) (n =8) *p*

Age (y)^a^ 70.0 ± 2.0 69.8 ± 1.4 68.3 ± 2.4 0.791

Sex (M/F)^b^ 2/6 3/9 2/6 1.000

BMI (kg/m^2^)^a^ 20.5 ± 0.6 20.9 ± 1.0 20.2 ± 0.8 0.830

Education (y)^a^ 12.6 ± 0.9 12.8 ± 0.4 13.0 ± 0.9 0.939

MoCA-J^a^ 20.9 ± 1.1 23.5 ± 0.8 23.0 ± 1.0 0.152

WMS-R LM II^a^ 14.5 ± 1.1 11.3 ± 2.0 14.3 ± 2.1 0.381

LCPUFA in plasma PL

ARA (%)^a^ 9.5 ± 0.5 9.2 ± 0.3 10.1 ± 0.5 0.425

EPA (%)^a^ 2.2 ± 0.3 2.6 ± 0.4 2.2 ± 0.7 0.728

DHA (%)^a^ 8.5 ± 0.6 8.3 ± 0.5 7.6 ± 0.7 0.588

SMI (kg/m^2^)^a^ 5.5 ± 0.2 5.6 ± 0.2 5.4 ± 0.3 0.900

Physical activity (METs/day)^a^ 1.0 ± 0.1 0.9 ± 0.1 1.0 ± 0.0 0.144

Step (/day)^a^ 5,473 ± 545 5,969 ± 596 6,841 ± 1335 0.561

Mean ± SE. There was no significant difference among the groups in baseline data (^a^ANOVA, ^b^chi-square test). Ex, exercise; BMI, body mass index; MoCA-J, Montreal Cognitive Assessment Japanese version; WMS-R LM II, Wechsler memory scale-revised logical memory II; LCPUFA, long-chain polyunsaturated fatty acid; PL, phospholipids; ARA, arachidonic acid; EPA, eicosapentaenoic acid; DHA, docosahexaenoic acid; SMI, skeletal muscle mass index; AWGS, the sarcopenia cut-off value in Asian Working Group for Sarcopenia.

**Supplementary Table S4. Muscle mass, physical activity and fatty acid composition in plasma phospholipid in the groups during the intervention in subgroup analysis by SMI < AWGS**

Group Baseline 24 weeks Δ

Muscle mass (kg) no Ex + placebo 31.8 ± 1.6 31.4 ± 1.6 -0.3 ± 0.2

Ex + placebo 32.2 ± 2.0 32.6 ± 2.1 0.3 ± 0.2

Ex + LCPUFA 31.8 ± 2.0 32.0 ± 2.1 0.1 ± 0.2

Step (/day) no Ex + placebo 5,473 ± 545 5,553 ± 604 81 ± 440

Ex + placebo 5,969 ± 596 7,055 ± 539^*^ 1,085 ± 400

Ex + LCPUFA 6,841 ± 1335 7,686 ± 889 845 ± 532

FA composition in plasma PL

PA (%) no Ex + placebo 26.7 ± 0.4 26.5 ± 0.6 -0.2 ± 0.5

Ex + placebo 26.4 ± 0.3 26.5 ± 0.3 0.1 ± 0.2

Ex + LCPUFA 26.6 ± 0.2 26.4 ± 0.3 -0.2 ± 0.2

SA (%) no Ex + placebo 14.7 ± 0.4 14.7 ± 0.5 0.0 ± 0.3

Ex + placebo 14.8 ± 0.2 14.5 ± 0.3 -0.2 ± 0.1

Ex + LCPUFA 14.3 ± 0.2 14.6 ± 0.4 0.3 ± 0.2

OA (%) no Ex + placebo 9.4 ± 0.3 8.9 ± 0.1 -0.5 ± 0.3

Ex + placebo 9.5 ± 0.2 9.0 ± 0.3 -0.5 ± 0.3

Ex + LCPUFA 9.5 ± 0.4 8.9 ± 0.2 -0.6 ± 0.3

LA (%) no Ex + placebo 19.2 ± 1.0 18.6 ± 1.1 -0.6 ± 0.6

Ex + placebo 19.7 ± 1.0 19.3 ± 0.9 -0.4 ± 0.8

Ex + LCPUFA 20.0 ± 1.0 18.7 ± 0.8^*^ -1.2 ± 0.5

ARA (%) no Ex + placebo 9.5 ± 0.5 9.6 ± 0.6 0.1 ± 0.3

Ex + placebo 9.2 ± 0.3 9.1 ± 0.3 -0.1 ± 0.4

Ex + LCPUFA 10.1 ± 0.5 10.8 ± 0.6^*^ 0.8 ± 0.2

EPA (%) no Ex + placebo 2.2 ± 0.3 2.4 ± 0.4 0.2 ± 0.4

Ex + placebo 2.6 ± 0.4 3.2 ± 0.5 0.6 ± 0.5

Ex + LCPUFA 2.2 ± 0.7 2.1 ± 0.3 -0.1 ± 0.7

DHA (%) no Ex + placebo 8.5 ± 0.6 8.7 ± 0.7 0.2 ± 0.4

Ex + placebo 8.3 ± 0.5 8.4 ± 0.6 0.1 ± 0.4

Ex + LCPUFA 7.6 ± 0.7 8.7 ± 0.3^*^ 1.1 ± 0.4

Mean ± SE. no Ex + placebo (n = 8), Ex + placebo (n = 12) and Ex + LCPUFA (n = 8) groups. There was no significant difference among the groups for each fatty acid, muscle mass and step at baseline (one-way ANOVA). There was no significant difference in change (Δ) between the either Ex groups and the no Ex + placebo (Dunnett's). ^*^*p* < 0.05 vs. baseline (paired *t*-test). Ex, exercise; LCPUFA, long-chain polyunsaturated fatty acids; FA, fatty acid; PL, phospholipid; PA, palmitic acid; SA, stearic acid; OA, oleic acid; LA, linoleic acid; ARA, arachidonic acid; EPA, eicosapentaenoic acid; DHA, docosahexaenoic acid; SMI, skeletal muscle mass index; AWGS, the sarcopenia cut-off value in Asian Working Group for Sarcopenia.

**Supplementary Table S5. Neuropsychological tests in the groups during the intervention in subgroup analysis by SMI < AWGS**

Group Baseline 24 weeks Change (Δ) *r* Δ adjusted *r*

**Attention**

Selective

Stroop CW step 1 no Ex + placebo 50.5 ± 2.6 48.5 ± 4.0 -2.0 ± 2.0 - -2.0 ± 1.9 -

Ex + placebo 50.7 ± 3.0 52.5 ± 2.8 1.8 ± 1.1 0.39 1.8 ± 1.5 0.40

Ex + LCPUFA 46.5 ± 3.4 50.8 ± 4.2 4.3 ± 2.3^#^ 0.48 4.3 ± 1.9^#^　 0.52

Stroop CW step 3 no Ex + placebo 33.5 ± 2.1 33.9 ± 2.4 0.4 ± 0.5 - 0.4 ± 0.9　　 -

Ex + placebo 34.2 ± 2.5 34.5 ± 2.4 0.3 ± 0.6 0.01 0.5 ± 0.8 0.00

Ex + LCPUFA 30.8 ± 3.1　 32.0 ± 2.7 　 1.3 ± 1.5　 0.15 1.0 ± 0.9　 0.10

Selective/divided

TMT-A no Ex + placebo 35.1 ± 3.3 37.9 ± 2.9 2.7 ± 2.8 - 2.8 ± 3.6 -

Ex + placebo 32.7 ± 2.5 30.0 ± 2.3 -2.7 ± 1.8 0.38 -3.1 ± 2.9 0.48

Ex + LCPUFA 37.1 ± 3.9 36.0 ± 6.8 -1.1 ± 5.5 0.17 -0.6 ± 3.6　 0.15

Divided

TMT-B no Ex + placebo 86.3 ± 11.8 91.0 ± 12.4 4.7 ± 15.1 - 6.2 ± 11.2 -

Ex + placebo 80.0 ± 9.6 74.0 ± 6.9 -6.1 ± 5.1 0.18 -7.1 ± 9.1 0.29

Ex + LCPUFA 82.9 ± 8.5 79.8 ± 17.8 -3.1 ± 14.4 0.10 -3.0 ± 11.2　 0.12

**Working memory**

Digit Span no Ex + placebo 11.8 ± 1.6 10.4 ± 1.6 -1.4 ± 0.9 - -1.1 ± 0.8 -

Ex + placebo 10.6 ± 0.8 10.8 ± 0.9 0.3 ± 0.5 0.37 0.2 ± 0.6 0.35

Ex + LCPUFA 10.1 ± 1.6 12.5 ± 1.0 2.4 ± 1.1^##^ 0.59 2.2 ± 0.8^#^　 0.59

**Executive function**

Inhibitory control

Stroop CW step 2 no Ex + placebo 39.6 ± 3.2 41.1 ± 4.0 1.5 ± 2.4 - 0.9 ± 2.0 -

Ex + placebo 45.8 ± 2.4 46.8 ± 2.7 1.0 ± 1.2 0.05 1.9 ± 1.7 0.06

Ex + LCPUFA 39.8 ± 5.0 42.4 ± 3.0 2.6 ± 2.6 0.08 2.0 ± 2.0　 0.11

Stroop CW step 4 no Ex + placebo 21.3 ± 5.1 23.8 ± 4.1 2.5 ± 4.6 - 1.0 ± 2.8 -

Ex + placebo 28.4 ± 4.0 27.8 ± 4.0 -0.7 ± 1.4 0.18 0.1 ± 2.3 0.07

Ex + LCPUFA 27.0 ± 3.7　 27.0 ± 2.4 　 0.0 ± 2.6　 0.12 0.3 ± 2.7　 0.05

Cognitive flexibility

KWCST CA no Ex + placebo 2.6 ± 0.8 2.3 ± 0.7 -0.4 ± 0.7 - -0.6 ± 0.6 -

Ex + placebo 3.3 ± 0.5 3.3 ± 0.6 -0.1 ± 0.6 0.07 0.0 ± 0.5 0.18

Ex + LCPUFA 3.5 ± 0.8 3.4 ± 0.8 -0.1 ± 0.3 0.09 0.0 ± 0.6　 0.20

Language flexibility

Verbal fluency no Ex + placebo 65.0 ± 3.9 65.0 ± 4.7 0.0 ± 3.8 - -0.6 ± 3.5 -

Ex + placebo 66.2 ± 4.9 70.3 ± 4.4 4.2 ± 3.2 0.19 3.8 ± 2.9 0.23

Ex + LCPUFA 76.0 ± 8.7　 73.5 ± 8.8 　 -2.5 ± 2.9　 0.14 -1.3 ± 3.6　 0.09

**Episodic memory**

Verbal immediate

WMS-R LM I no Ex + placebo 20.1 ± 1.5 19.1 ± 1.6 -1.0 ± 1.8 - -0.8 ± 2.2 -

Ex + placebo 17.5 ± 1.9 20.1 ± 1.5 2.6 ± 1.4 0.35 2.2 ± 1.8 0.28

Ex + LCPUFA 20.9 ± 1.9 23.1 ± 4.1 2.3 ± 3.0 0.24 2.6 ± 2.2　 0.24

Verbal delayed

WMS-R LM II no Ex + placebo 14.5 ± 1.1 17.0 ± 1.7 2.5 ± 1.6 - 2.6 ± 2.1 -

Ex + placebo 11.3 ± 2.0 15.8 ± 2.0^**^ 4.6 ± 1.4 0.22 4.4 ± 1.8 0.12

Ex + LCPUFA 14.3 ± 2.1　 19.6 ± 4.0 　 5.4 ± 2.8　 0.23 5.5 ± 2.1　 0.24

Visual delayed

ROCFT recall no Ex + placebo 12.6 ± 1.5 17.5 ± 2.5 4.9 ± 2.4 - 4.3 ± 2.0 -

Ex + placebo 12.8 ± 2.2 13.9 ± 1.6 1.1 ± 2.0 0.28 0.5 ± 1.6 0.32

Ex + LCPUFA 17.1 ± 2.3　 15.3 ± 2.3 　 -1.8 ± 1.7　 0.52 -0.2 ± 2.0　 0.42

Mean ± SE. no Ex + placebo (n = 8), Ex + placebo (n = 12) and Ex + LCPUFA (n = 8) groups.　One participant’s data in the Ex + placebo group was lacked in Stroop CW step 2. There was no significant difference among the groups for each fatty at baseline (one-way ANOVA).　^#^*p* < 0.05 and ^##^*p* < 0.01 vs. the no Ex + placebo group (Dunnett's). ^**^p < 0.01 vs. baseline (paired *t*-test).　Change adjusted by baseline score was expressed as Δ adjusted　Ex, exercise; LCPUFA, long-chain polyunsaturated fatty acids; Stroop CW, Stroop Colour-Word; TMT, Trail making test; KWCST CA, Wisconsin card sorting test of Keio version category achieved; WMS-R LM I/II, Wechsler memory scale revised logical memory I/II; ROCFT, Rey-Osterrieth complex figure test; SMI, skeletal muscle mass; AWGS, the sarcopenia cut-off value in Asian Working Group for Sarcopenia index. Effect size is expressed as *r*.
